# Supplementary material for: Intralocus sexual conflict can resolve the male-female health-survival paradox
Source: Nat Commun. 2018 Nov 28;9:5048. doi: 10.1038/s41467-018-07541-y (PMC6261961; doi:10.1038/s41467-018-07541-y)
Supplement: Supplementary file 1 — Supplementary Information [file 41467_2018_7541_MOESM1_ESM.pdf]

SUPPLEMENTARY INFORMATION

Intralocus sexual conflict can resolve the male-female health-survival paradox

Archer *et al.*

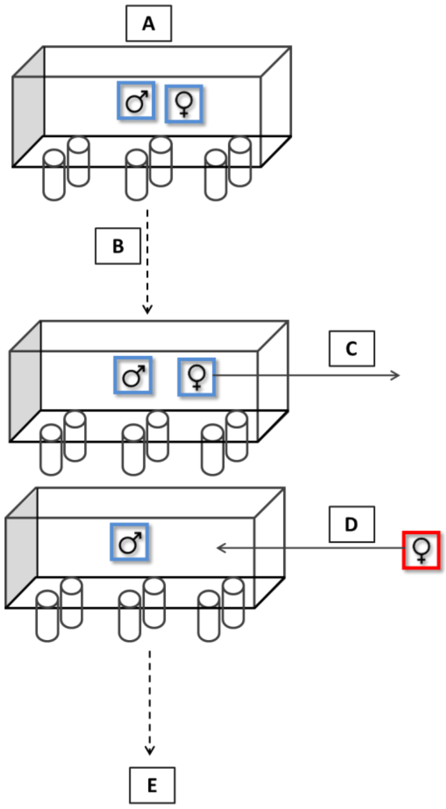

Supplementary Figure 1. Selection Regime. Male and female symbols represent 100 flies. Symbols outlined in blue represent flies from an experimental population, symbols outlined in red represent flies from paired “feeder” cages that provides virgin female mates – note, each experimental population has an independent feeder cage. The cages used in this experiment (11 x 16 x 31 cm) have mesh at one side (shaded) to allow flies to be introduced or removed. Each cylinder represents an individual 60mL food vial. (**A**) 100 male and 100 female newly eclosed virgins are introduced to the cage. (**B**) Over the next 28 days, flies are given new food every 5 days, to ensure that offspring do not crawl up and pupate in the cage. (**C**) On the 28th day, all flies are gassed with $C0_2$, males are retained while females are removed. (**D**) 100 virgin females are introduced into the cage. These were collected 3-5 days beforehand and kept in 40mL vials with 8mL of food in groups of 6. Fresh food is provided. (**E**) Three days later food is removed, incubated and offspring collected for the next generation.

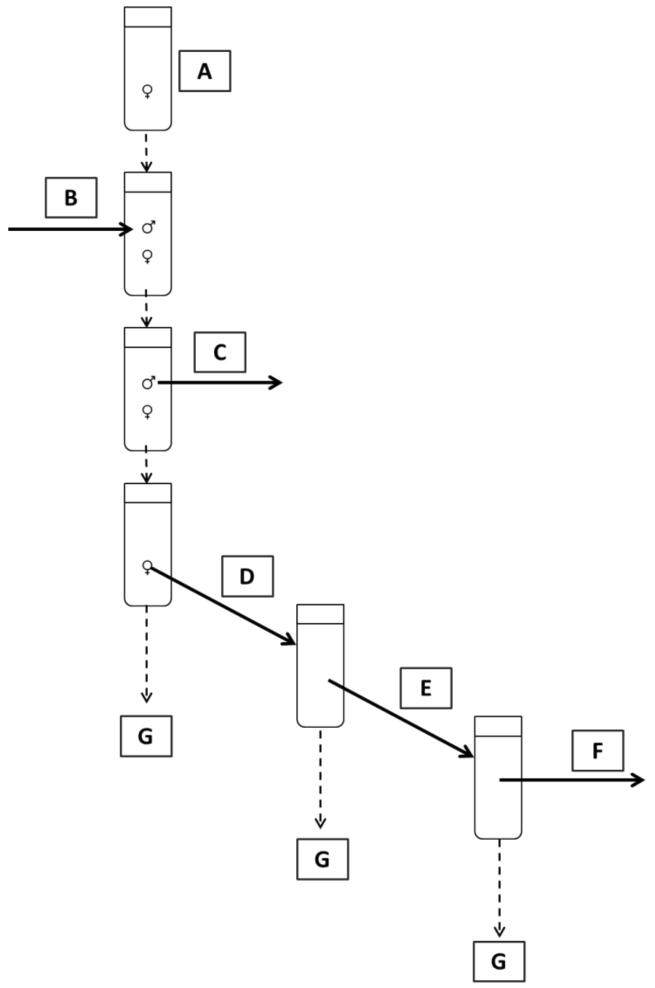

Supplementary Figure 2. Male fertility assay for experimentally evolved populations. Dashed lines represent the incubation of a vial, solid lines represent the movement of a fly. Male and female symbols represent a single fly. (**A)** An individual stock virgin female fly (3-6 days old) was added to a 40mL vial with 8mL of food at around 17:00. (**B**) At 09:00 the following day, a focal assay male (28 days old) was added. (**C**) Males were removed at 17:00 hours. (**D**) Females were allowed 3 days to lay in this vial before being moved to fresh vial for another two days and (**E)** then finally to a third vial for two more days before being removed (**F**). (**G**) Once females were removed from vials, vials were monitored daily. Once a fly emerged from food, vials were incubated for one week and then all offspring were counted. Male fertility is the sum of counts from each of these three vials, unsuccessful mating where no offspring are produced are excluded from analyses.

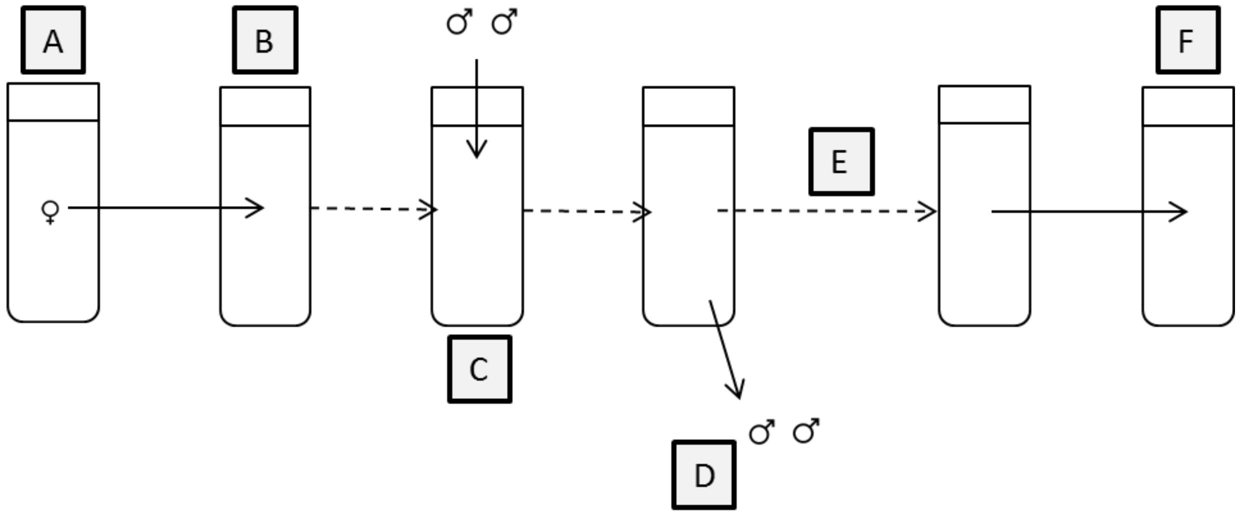

Supplementary Figure 3. Assaying female lifespan in experimentally evolved populations. Solid lines represent the movement of a fly, dashed lines represent the incubation of vials. Male and female symbols represent a single fly and vials are all 40mL and contain 8mL of food. (**A**) A virgin, newly eclosed female was introduced to a vial. (**B)** Females were left for four days and then moved to a clean vial between 17:00 and 18:00 hours. (**C)** The following morning, two virgin stock males (between 3 and 6 days old) were introduced and then removed three hours later (**D)**. (**E**) Females were left for four days before being moved to a clean vial. (**F)** Return to step C. **Throughout:** Female survival was checked daily and if a vial became slimy due to bacterial growth, females were moved to a fresh vial.

Supplementary Table 1. Sample sizes for assays of male fertility and female health in DRGP lines. 8 males were collected from each line to measure male fertility and 8 females from each line were randomly allocated to one of the assays (i.e. geotaxis distance or recovery time) and reared individually until day 35. Randomisation was achieved using a random number generator online. However, poor survival meant that for some lines too few animals survived for us to be able to calculate a reasonable line mean. The lines in the table below all had ≥ 2 animals surviving in at least two of the assay groups. Derived variables were calculated from these measures for regression analyses as described in the main manuscript. Please note, line 783 was excluded from the regression of male fertility and recovery time, because too few females survived to assay recover time. This left the following sample sizes. NB – this table includes males that may not have mated and generated fertility values of zero.

Line ID	Male Fertility	Recovery Time	Geotaxis Distance
28	6	5	2
101	2	6	2
136	6	5	6
360	8	5	5
595	5	6	5
737	7	6	6
783	5	NA	5
796	6	5	3
